# Supplementary material for: Mood disorders in children following neonatal hypoxic-ischemic encephalopathy
Source: PLoS One. 2022 Jan 28;17(1):e0263055. doi: 10.1371/journal.pone.0263055 (PMC8797213; doi:10.1371/journal.pone.0263055)
Supplement: S1 File — (PDF) [file pone.0263055.s001.pdf]

GROUP	GEST.AGE	NWB.WEIGHT	EDU.MAT	EDU.PAT	INCOME	AGE@EXPL
CTL	39	3230	3	3	6	3
CTL	37	3010	7	7	6	5
CTL	40	3100	7	7	6	5
CTL	40	3500	7	7	6	4
CTL	39	3400	7	7	6	5
CTL	42	4255	4	7	6	5
CTL	38	2475	7	7	6	5
CTL	40	3160	7	7	6	5
CTL	41	4270	7	7	4	5
CTL	40	3650	7	7	6	4
CTL	35	2000	6	7	6	4
CTL	35	1950	6	7	6	5
CTL	39	3500	7	7	6	5
CTL	40	3210	7	7	6	3
CTL	38	3100	7	7	6	4
NHIE	39	2790	7	7	6	5
NHIE	38	3080	7	7	6	5
NHIE	38	3000	7	7	6	5
NHIE	40	2480	5	2	5	3
NHIE	40	4000	7	7	6	4
NHIE	39	3500	7	7	6	4
NHIE	40	3500	7	7	4	3
NHIE	37	2850	4	7	5	4
NHIE	37	2950	7	7	6	4
NHIE	39	3000	7	7	6	3
NHIE	35	2900	7	7	5	5
NHIE	38	4500	3		4	3
NHIE	41	3380	6	3		5
NHIE	39	3100	6	6	6	5
	weeks	grams	1 =NO	1 =NO	1 = Charity	years
			2 = 1st school	2 = 1st school	2 = unemplyment assist	
			3 = 2nd school	3 = 2nd school	3 = 480 – 1000€/month	
			4 = professional	4 = professional	4 = 1000 – 1500€	
			5 = High school	5 = High school	5 = 1500 – 2000€	
			6= Specialized p	6= Specialized p	6 = >2000€	
			7= University	7= University		

PRESS	GROUP	ASQ3_Comun	ASQ3_MotGros	ASQ3_MotFine	ASQ3_Resol	ASQ3_Soc
0	CTL	50	60	55	60	55
7	CTL	50	60	50	50	30
0	CTL	60	60	55	55	55
0	CTL	50	60	60	60	60
0	CTL	55	60	55	60	60
0	CTL	50	60	60	60	60
0	CTL	55	60	60	60	60
1	CTL	50	60	60	60	60
1	CTL	50	60	60	60	60
1	CTL	45	60	60	60	50
1	CTL	60	55	50	60	60
0	CTL	60	55	50	60	60
0	CTL	60	60	50	60	60
0	CTL	55	60	60	60	60
1	CTL	55	55	55	60	55
2	NHIE	55	60	55	60	60
0	NHIE	55	60	55	60	55
0	NHIE	50	60	60	60	60
6	NHIE	40	55	60	60	40
0	NHIE	50	50	35	50	35
2	NHIE	40	50	50	50	50
0	NHIE	45	55	45	55	40
4	NHIE	50	50	40	60	45
2	NHIE	45	55	60	50	50
3	NHIE	35	55	40	55	45
7	NHIE	45	60	40	60	55
7	NHIE	60	40	20	40	50
3	NHIE	20	60	40	45	50
4	NHIE	35	50	55	60	55
points		points	points	points	points	points

GROUP	cbcl1_5_React	cbcl1_5_Ans/Depr	cbcl1_5_Compla	cbcl1_5_Retrac	cbcl1_5_Sleep	cbcl1_5_Atent	cbcl1_5_Agress	cbcl1_5_Other	MDRS_Mother
CTL	3	6	1	0	6	6	15	12	3
CTL	2	0	0	1	5	4	12	6	4
CTL	2	1	2	2	2	3	6	9	2
CTL	6	1	0	3	2	4	12	9	1
CTL	3	3	1	4	0	4	7	15	2
CTL	1	1	2	1	2	0	4	8	2
CTL	0	0	4	0	0	3	5	1	2
CTL	4	3	3	1	5	2	17	11	10
CTL	2	2	0	2	1	4	5	4	3
CTL	1	5	2	0	6	2	5	3	1
CTL	0	0	1	3	0	2	5	8	3
CTL	0	0	2	1	0	1	5	2	3
CTL	0	2	2	1	0	0	1	3	1
CTL	0	0	0	0	1	1	0	3	2
CTL	2	2	0	1	2	0	4	3	1
NHIE	0	2	0	0	2	1	6	2	1
NHIE	0	2	3	0	0	2	8	1	2
NHIE	1	3	0	0	0	0	10	7	0
NHIE	13	9	5	8	4	6	30	27	15
NHIE	11	9	2	5	6	7	22	23	5
NHIE	8	7	3	7	0	3	6	17	10
NHIE	1	2	6	0	4	3	15	11	7
NHIE	2	1	2	0	5	3	6	5	3
NHIE	2	2	2	1	3	0	17	7	2
NHIE	0	1	1	1	1	2	12	3	1
NHIE	2	3	0	0	5	5	19	11	1
NHIE	2	2	0	0	0	3	11	3	16
NHIE	0	2	0	2	3	2	8	9	14
NHIE	1	1	2	0	2	3	5	7	16
	points	points	points	points	points	points	points	points	points
