# Supplementary material for: Mood disorders in children following neonatal hypoxic-ischemic encephalopathy
Source: PLoS One. 2022 Jan 28;17(1):e0263055. doi: 10.1371/journal.pone.0263055 (PMC8797213; doi:10.1371/journal.pone.0263055)
Supplement: Attachment — Submitted filename: Response to Reviewers.docx [file pone.0263055.s002.docx]

PONE-D-21-13777

Answers to Reviewer #1:

We wish to thank the Reviewer because his/her comments have greatly improved the quality of the manuscript.

Comments:

- *I will recommend the authors to include more clearly the number of preterm and term infants in the study, since the mean age in both groups was 38 weeks.*
  - This has been included in the revised manuscript (lines 194-195). Since the original statement was confusing -all babies were of more than 35 weeks of gestational age-, it has been rewritten (line 107).
- *My recommendation is to use the complete title of the tests also in the abstract.*
  - Complete title of tests have been included in the Abstract
- *Magnetic Resonance Images (MRI) were obtained from the children early after birth and at the age of two to three years. Authors only described the results of the images, but not include any figure. I think that the presentation of the images of the child with a lesion at birth and that was normal after 3 years will be welcome. The authors only describe MRI studies of the HIE group. To have a perfect mismatch with the HIE children, studies of the healthy children should be also included. It has been shown by MRI (Rutherford et al., 2010) that hypothermia treatment of infants with HIE decreases brain injury tissues, therefore should be interesting to know the MRI results of the control group. In case of preterm infants frequent MRI abnormalities have been described (Volpe, J in 2011, 2019; Woodward et al. 2012; and Harmony, 2021).*
  - Representative examples of abnormal MRI in NHIE newborns with the correspondent normal scan when aged 2-3 years have been included in the revised version of the manuscript (Figure 2) (lines 182-189).
  - The aim of the present work was focused on the study of Mood Disorders in the hypoxic-ischemic babies. The aim of presenting the MRI studies was to explore the possibility that mood disorders were related to the presence of damage in specific brain regions, which was not the case. Our work was not designed to explore the effects of hypothermia on hypoxic-ischemic brain damage as assessed by MRI, which has been extensively studied already, as the Reviewer stated. That is why the Ethical Committee did not approve to perform MRTI studies on healthy babies and children, who should have been exposed to sedation for the MRI study to be performed.
  - MRI abnormalities are present mostly in very preterm infants (under 32 weeks of gestational age), as described in the bibliography cited by the Reviewer. Thus, the American Association of Neurology recommends since 2004 that routine MRI studies were performed only in very preterm infants or in those newborns at high neurological risk (Ment L et al, 2004). In our case, the only baby born at 36 weeks of gestational age in the NHIE group showed no abnormalities at the MRI studies.

PONE-D-21-13777

Answers to Reviewer #2:

We wish to thank the Reviewer because his/her comments have greatly improved the quality of the manuscript.

Comments:

- *Results should be presented by medians instead of means*
  - All results have been presented as medians in tables and text in the revised manuscript
- *I fully understand that it is difficult to blind the assessors in a clinical setting, but I really miss discussion how not-blinded evaluation of the outcome affects the results.*
  - The reviewer points out a very relevant aspect of clinical research. Having into account that NHIE children were assessed at the follow-up clinical whereas healthy children attended a special visit, blinding was impossible. However, the different measures collected in our study were either completed by parents or children (therefore without the intervention of the evaluator) or are highly structured and, again, do not require the interpretation of the researcher. Thus, Ages and Stages Questionnaire 3 (ASQ-3) and Child Behavior Checklist (CBCL) are questionnaires answered by parents, whereas the Preschool Symptom Self-Report (PRESS) scores as “present” or “absent” the answers of the child to a series of drawings. The Montgomery-Asberg Depression Rating Scale (MADRS) is a self-reported questionnaire. Therefore, researcher’s knowledge of about the experimental group is unlikely to interfere with the score since this was independent from the researcher’s opinion. This, lack of blinding is usual in this kind of studies (please see refs. 11 and 12 of the manuscript). This has been included in the Discussion (lines 441-447).
- *Line 69-71. The authors state that emotional consequences of disability of CP can mask the effects of NHIE by itself. I totally agree. Later, when the authors cite the same study in the discussion, Line 287, they interpret results differently than the authors of the cited study. Rackauskaite et al. wrote “the high prevalence of psychopathology in children with CP may be due to brain impairment or cognitive disability and not to the motor disability itself”. Please, correct it.*
  - This has been corrected in the manuscript (lines 386-387).
- *Line 160-164 presents data from the Figure 1. There is no need for repetition*.
  - This has been so corrected in the revised version of the manuscript (line 170)
- *Line 182/ Table 1. There is a difference of 6 months between the mean age of control and NHIE. Emotional development is quite fast between the age of 4½ and 4 years. Please, discuss if that can influence your results, even the ages are not statistically significant.*
  - Besides the lack of statistical difference respecting the age at evaluation, age rank in both populations was very similar. In addition, CBCL 1.5-5 applies the same questionnaire for children between 1.5 and 5 years of age, assuming that responses between those boundaries are not affected by age (please see ref. 15 of the manuscript). In the case of ASQ-3 and PRESS, these are standardized tests, i.e., normalized for age. Therefore, the influence of the age difference in the results of our study is unlikely. In fact, even broader age ranks can be found in studies similar to ours (please see refs 11 and 12 of the manuscript).
- *Line 238 is difficult to read. Please, rephrase.*
  - The line has been rephrased in the revised version of the manuscript (lines 338-339)
- *Line 270-275 repeats information from the introduction.*
  - Repeated information has been removed from the Introduction in the revised version of the manuscript (line 87).
- *Line 332-334. Handicap is a strong word in the context of HNIE. I suggest to reword “could be a handicap” by using a verb like “could disturb” or “interfere with”.*
  - This has been so reworded in the revised version of the manuscript (line 439)
- *Line 341-342. Your data does not support the statement, that MD were not related to the severity of handicap. All children (HNIE and controls) had a normal motor and cognitive function, thereby no handicap.*
  - A more appropriate statement has been included in the revised version of the manuscript (lines 454-455)
